# Psychedelic Cognition—The Unreached Frontier of Psychedelic Science

**DOI:** 10.3389/fnins.2022.832375

**Published:** 2022-03-15

**Authors:** Maria Bălăeţ

**Affiliations:** Department of Brain Sciences, Imperial College London, London, United Kingdom

**Keywords:** psychedelic, cognition, memory, attention, reasoning, creativity, social cognition

## Abstract

Psychedelic compounds hold the promise of changing the face of neuroscience and psychiatry as we know it. There have been numerous proposals to use them to treat a range of neuropsychiatric conditions such as depression, anxiety, addiction and PTSD; and trials to date have delivered positive results in favor of the novel therapeutics. Further to the medical use, the wider healthy population is gaining interest in these compounds. We see a surge in personal use of psychedelic drugs for reasons not limited to spiritual enhancement, improved productivity, aiding the management of non-pathological anxiety and depression, and recreational interests. Notably, microdosing—the practice of taking subacute doses of psychedelic compounds—is on the rise. Our knowledge about the effects of psychedelic compounds, however, especially in naturalistic settings, is still fairly limited. In particular, one of the largest gaps concerns the acute effects on cognition caused by psychedelics. Studies carried out to date are riddled with limitations such as having disparate paradigms, small sample sizes, and insufficient breadth of testing on both unhealthy *and* healthy volunteers. Moreover, the studies are majoritarily limited to laboratory settings and do not assess the effects at multiple dosages within the same paradigm nor at various points throughout the psychedelic experience. This review aims to summarize the studies to date in relation to how psychedelics acutely affect different domains of cognition. In the pursuit of illuminating the current limitations and offering long-term, forward-thinking solutions, this review compares and contrasts findings related to how psychedelics impact memory, attention, reasoning, social cognition, and creativity.

## Introduction

Psychedelic drugs are making a strong come-back in the research, clinical, and public spheres. Studies carried out in the past two decades suggest psychedelic drugs as potential therapeutics for depression (Carhart-Harris et al., 2016a), anxiety (Gasser et al., 2014), substance use disorders (de Veen et al., 2017) such as tobacco addiction (Johnson et al., 2017) or alcoholism (Bogenschutz et al., 2015), post-traumatic stress disorder (Krediet et al., 2020), obsessive-compulsive disorder (Moreno et al., 2006), anorexia (Foldi et al., 2020), and inflammatory syndromes (Flanagan and Nichols, 2018). Promising results are giving patients hope of relieving the burden inflicted by their conditions. These results are further inspiring worldwide interest, with numerous studies being conducted (Siegel et al., 2021), psychedelic therapy clinics opening up (Doblin et al., 2019; Rucker and Young, 2021), and therapists being trained to work with psychedelic substances (Holoyda, 2020). Outside the scientific and psychiatric setting psychedelics are promoted as cognitive enhancers, spiritual catalysts, and general wellbeing aids. Microdosing—the practice of taking allegedly subacute doses every few days in order to enhance creativity, productivity, and wellbeing—is of increasingly greater interest amongst professionals working in office jobs and in the creative industries alike (Kuypers et al., 2019; Bornemann, 2020; Hutten et al., 2020; Askew and Williams, 2021).

The 1962 amendment on psychoactive substances only being allowed on the market upon proof of efficacy being established through controlled clinical trials adversely affected the methodological breadth of psychedelics research (Oram, 2014). In 1970 psychedelic substances were placed under Schedule I based on the United States Controlled Substances Act, and as a consequence research, as well as personal use, almost stopped (Belouin and Henningfield, 2018). Today there is a shift in the discourse; concerning both medical and personal use, decriminalization and/or legalization are on the horizon—or realities, even—in some countries (Pellegrini et al., 2013). Clinical studies have so far had overwhelmingly positive outcomes. Moreover, previous work has illustrated that they are neither neurotoxic nor addictive (Meyer and Maurer, 2011; Rucker et al., 2018). Relaxation of laws and a decrease in social stigma associated with psychedelics has resulted in not only easier routes for research and increased access to therapy but also a spike in recreational use. There is a growing trend around recreational consumption of substances such as LSD (Yockey et al., 2020) or DMT (Palamar and Le, 2018), which might be, in part, due to public perception that psychedelics are safe for personal use. Though scientists hypothesized that psychedelics could potentially act as cognitive enhancers in the case of major depression (Magaraggia et al., 2021), such links have not been formally drawn regarding non-clinical populations, who are using these substances increasingly more. At the societal and individual level, perception of the safety, utility, and everyday role of psychedelics has changed dramatically. This change is generally perceived to be a positive one. But now the question should be: are we moving too fast? Psychedelics are notorious for inducing “ego dissolution” and transporting people to seemingly different realms. The aforementioned changes in law are being driven primarily by generalizations based upon rather small laboratory-based studies, where extra care has been dedicated to selecting participants, and also assisting them during their experiences. Notably, it is documented in the literature how important it is that users be assisted by a trained professional during their trip (Phelps, 2017). But these circumstances are extremely different from the typical circumstances in which people consume psychedelics recreationally. Therefore, there is an urge to gather evidence from large empirical studies looking at the effects of psychedelics in *naturalistic settings* in order to inform harm reduction measures, and subsequently, policies.

One of the most neglected aspects in the research of psychedelics is their effect on cognition and its distinctive domains. A recent systematic review and meta-analysis looking at the neuropsychological functioning in users of serotonergic psychedelics only included 13 studies (Basedow et al., 2021). So far most of the efforts have been centered around mental health outcomes (De Gregorio et al., 2021), effects on brain activity (Carhart-Harris et al., 2016b), and guidelines for therapy (Holoyda, 2020)---but not substantially and specifically on acute and long-term impact on cognitive function. The largest gap in knowledge refers to how psychedelics affect cognition acutely. It is critical to understand how cognition is affected by psychedelics and what factor parameters make psychedelics experiences either positive or negative. In the clinical setting, such as in the case of psychedelic-assisted therapy, understanding cognition is key to the treatment process itself, since the modulation of various cognitive domains would shape what the participant can or cannot engage into. It is worth noting, however, that in the case of psychedelic assisted therapy, the dosage and purity of the drugs are highly controlled and the amount of times the drugs are administered is limited. Outside the clinical setting, where there are a lot more variables to account for---purity, dosage, frequency of use, and more---effects on cognition are a matter of safety for the participant. When cognitive processes are affected acutely, as in neuropsychiatric conditions, patients require constant monitoring and assistance because the risk of them endangering themselves or others is significantly increased. This review aims to summarize existing research in the psychedelics field assessing the acute effects of psychedelics on human cognition, identify incongruent results, point out the limitations of studies to date, and provide guidance toward improving the current body of knowledge. The literature covered has been selected through a manual search carried out between April 2020 and August 2021 on the PubMed^1^ and Google Scholar^2^ databases for the terms “cognition psychedelics,” “memory psychedelics,” “attention psychedelics,” “reasoning psychedelics,” “creativity psychedelics,” and “social cognition psychedelics.” For each term, additional searches were made by substituting “psychedelics” with “LSD,” “psilocybin,” “DMT,” and “mescaline.” All articles found that were assessing the impact of psychedelics on cognition using cognitive tasks have been included. A summary of all findings discussed can be found in Supplementary Table 1.

## Understanding Cognition

Cognition is the ability to gather and process information via the senses in order to make sense of our environments and, ultimately, guide our behavior. It is a multifactorial process comprising several discrete cognitive domains that relate to key abilities, namely memory, attention, reasoning, language, and social cognition. The functioning of these cognitive domains is measurable with instruments known as *cognitive tasks*, which can be pen and paper based or computerized. However, certain higher order cognitive processes require the integration of multiple domains. Planning, for example, engages working memory as well as decision-making abilities. Another example which can simultaneously engage all cognitive domains is creative problem-solving. Isolating different cognitive domains for the purpose of measuring cognitive abilities and designing tasks that capture distinct aspects of cognition has proven to be challenging due to this synergistic overlap, which is why there are a number of distinct paradigms available to test the same aspects of cognition and a primary reason we observe heterogeneous results when testing the cognitive abilities of various populations. Nevertheless, understanding how different cognitive processes are affected by different states, such as the psychedelic state, is essential for identifying impairments and enhancements, and understanding the extent to which cognitive performance—under the influence of various drugs, at different stages of the experience, and in different settings—can cause harm, or conversely, provide benefits.

Classic psychedelic drugs such as lysergic acid diethylamide (LSD), psilocybin, N, N-dimethyltryptamine (DMT) and mescaline produce their profound effects on perception and cognition via the activation of the serotonergic 2A receptors (5-HT_2A_ receptors) (Howland, 2016; Nichols, 2016). The healthy functioning of the serotonergic system—specifically the 5-HT_2A_ receptor—has been documented in the literature as being essential for optimal cognition (Harvey, 2003; Zhang and Stackman, 2015). Whilst the effects of psychedelics on cognition have been studied *in vivo* in animals such as mice (Zhang et al., 2017), rats (Macúchová et al., 2017), rabbits (Romano et al., 2010), and monkeys (Frederick et al., 1997), this review focuses on human studies only to maintain an ecologically valid perspective.

Previous studies looking at how psychedelic drugs impact cognition have produced mixed results. Early studies looking at psychedelics such as LSD suggested that being under the influence makes it difficult to carry out cognitive tasks (Goldberger, 1966). Since then, a plethora of paradigms have been used to study these effects—but only on a couple of cognitive domains at a time, at different dosages and different timepoints during the psychedelic experience across different studies, and in different settings (primarily in the laboratory); there are only a few studies looking at effects of cognition under the influence for psychedelics in naturalistic environments (Bouso et al., 2013; Prochazkova et al., 2018). To exemplify the inconsistency of testing paradigms, in a study done by Barrett et al. (2018) three tasks part of the Penn Computerized Neurocognitive Battery (CNB) (Gur et al., 2010) were computerized, and one was administered verbally. Furthermore, another task administered (Stroop Task) was *not* part of the CNB paradigm and was added to extend the domains studied. So far there have been attempts only to synthesize the literature either on the effects of the drug (Dos Santos et al., 2016), or on specific cognitive domains only (Preller and Vollenweider, 2019; Rocha et al., 2019); there is no body of work attempting to integrate all knowledge of the effects on the multiple domains of cognition. These approaches are partly justified since psychedelics appear to cause specific impairments rather than acute global cognitive impairment, as seen on the Mini Mental State Examination (Barrett et al., 2018). However, prior research also illustrated that small psychedelic doses produce no effects on cognition at all (Bershad et al., 2019), which suggests that interpreting the effects of psychedelics on cognition is not that simple. The present review aims to go a step further and fill this gap by providing a synthesis of how psychedelic drugs affect each cognitive domain in turn, identify where conclusions are in contradiction, and point toward how these could be resolved in future studies.

## Memory

Memory is an essential cognitive process, fundamental to the process of learning and thus the brain’s adaptability to novel situations. Early work by Williams et al. (2002) has highlighted that the 5-HT_2A_ receptors play a key physiological role in working memory, and that alterations in their signaling could be underlying cognitive dysfunction in depression or schizophrenia, in turn making them attractive therapeutic targets for these conditions. Carter et al. (2005) who sought to investigate whether psilocybin effects on memory are mediated by the 5-HT_2A_ receptors, found no effects on spatial working memory upon psilocybin administration. Other studies, however, found that psilocybin does have an effect on memory, and more recent neuroimaging studies have shown activation of areas involved in memory following the administration of psychedelic drugs (Carhart-Harris et al., 2012; Kaelen et al., 2016). Thus, there is an open question regarding to which extent psychedelic drugs can influence memory function.

Working memory is the ability to hold information in memory in the short-term, and has been addressed by a few studies. Carter et al. (2005) found no effects of psilocybin on working memory in eight healthy volunteers using the Spatial Span task from CANTAB. Using the same task, Wittmann et al. (2007) found significant impairments in spatial working memory at medium and high doses of psilocybin after assessing 12 healthy volunteers under the influence. These differences in results coming from the same laboratory could be due to increasing the dose of psilocibyn from 215 to 250 μg/kg, but also due to a larger sample size. Bouso et al. (2013) found impairments to working memory (represented as high errors in Sternberg task) following ayahuasca exposure in 24 users. Barrett et al. (2018) looking at twenty healthy volunteers under psilocybin, found that it affects working memory by increasing response time on Letter N-back but has no impact on accuracy. Studying lower doses, Bershad et al. (2019) found no acute LSD microdose effects on working memory on the dual N-back task twenty healthy volunteers. Family et al. (2020) found no effect of microdosed LSD on the spatial working memory task from CANTAB after assessing 48 subjects. Other types of memory have been much more scarcely addressed. Barrett et al. (2018) and Family et al. (2020) found decreased free recall in 20 healthy volunteers following psilocybin administration found no effect of LSD microdoses on the pair associates learning (PAL) task from CANTAB in 48 subjects.

Previous studies suggest that working memory is unanimously impaired at higher doses, but unaffected at medium and lower doses of psychedelics. It is, however, important to note the following: the number of subjects has been very limited in all studies; the paradigms used have been different for different drugs, such as Sternberg task being used to assess working memory in ayahuasca administration (Bouso et al., 2013) and Spatial Span task from the CANTAB battery being used to assess psilocybin (Carter et al., 2005; Wittmann et al., 2007) and LSD (Family et al., 2020); and the doses have varied widely—from microdoses (Family et al., 2020) to high doses (Wittmann et al., 2007). Amongst the many remaining unanswered questions is that of how psychedelics affect memory in naturalistic settings. What are the implications of the effects of psychedelics on working memory with regards to how these substances are used to treat diseases? For example, psychedelics are proposed for treating PTSD but it is unclear whether they would have an effect on destabilizing maladaptive memories as per different reconsolidation paradigms (Fattore et al., 2018), or whether they would affect non-maladaptive memories recalled during therapy in any way. Further, there are uncertainties about the permanency of possible memory impairment, and whether the process of remembering something during a trip causes disruption of processes such as memory reconsolidation, and what this means for long-term memory integrity. On the other hand, could specific doses of psychedelics be used for aiding the recollection of autobiographical memories, such as in the case of repressed memories (Healy, 2021)? Or should be prescribed as a preventative measure against dementia, as Family et al. (2020) suggested? Would this be safe? Indeed psychedelics are showing great promise, but further studies are needed to address the above questions. If psychedelics can produce beneficial effects on memory, the exact cases, substances, doses, and conditions under which they can or should be used must be established. And this is just as important as identifying the people who would be at increased risk from consuming them.

## Attention

Attention is the behavioral process where an individual concentrates on a particular stimulus without interference from other stimuli. This ability is particularly important in learning. Attention allows a person to carry out tasks, be it making a simple cup of coffee or something more complex like driving through a busy road or debating philosophical questions with a colleague. Carter et al. (2005) found that pre-treatment with the 5-HT_2A_ antagonist ketanserin did not prevent impairments in attentional tracking caused by psilocybin administration, and suggested these might be mediated by the 5-HT_1A_ receptor instead.

Findings around how psychedelics impact attention are mixed. It has been overwhelmingly suggested that DMT impairs attention—specifically inhibition of return, which is an otherwise protective mechanism against distracting stimuli (Gouzoulis-Mayfrank et al., 2006; Daumann et al., 2008). It is important to note, though, that the studies have employed only 14–15 volunteers and have been carried out by the same team, which suggests replication by other teams with a higher number of volunteers would be required. Albeit not significantly, DMT also appeared to decrease startle magnitude in nine volunteers (Heekeren et al., 2007). Other studies illustrated that psilocybin impaired sustained attention in sixteen volunteers (Vollenweider et al., 2007), decreased the acoustic startle in sixteen volunteers (Quednow et al., 2012), and reduced attentional tracking in eight volunteers (Carter et al., 2005). Carter et al. (2005) suggested that impaired attentional tracking ability under the influence of psilocybin might be related to a lack of ability to suppress distracting stimuli, which is similar to the mechanism proposed by Gouzoulis-Mayfrank et al. (2006) for DMT. Contrary to the impairments observed at higher psilocybin and DMT doses, effects observed at low LSD doses suggest that attention was enhanced in the majority of the 24 healthy volunteers on the Psychomotor Vigilance Task (Hutten et al., 2020). This latter study also spotlights the importance of noting individual variations in the effects of drugs, and suggests more research is needed to explore to what extent psychedelics can have enhancing effects on cognition and what factors may drive these effects. However, Family et al. (2020) found no effect of LSD microdosing on attention in all 48 volunteers using the Rapid Visual Information Processing Task from CANTAB. This suggests that using different substances and different paradigms for assessing attention, as well as the sample sizes, might be responsible for the discrepancies in results which make drawing a general conclusion difficult.

Due to the strong implications of using these drugs in order to improve attention, understanding how psychedelics impact attention is paramount. The results put forward so far suggest that low doses of LSD might be attention-enhancing, whereas higher doses of DMT or psilocybin affect the ability to filter out distracting stimuli. To elucidate where the tipping point lies, more research is needed to understand dose dependency when it comes to the effects of psychedelics on attention under the influence of all substances mentioned. Knowledge accumulated so far is insufficient to discern whether attention enhancement properties are drug-specific, or low doses of DMT or psilocybin would also behave in a manner similar to LSD. Whilst improved attention is an attractive life enhancing prospect in healthy people, there is also the question of whether psychedelics could be beneficial for people suffering with conditions such as dementia, TBI, ADHD etc., or for preventing Alzheimer’s, as Family et al. (2020) suggested. And then there are questions about whether the effects on attention can be sustained after the acute dose wears off, and if so, for what duration; and how removal of the drug from the treatment course would affect natural abilities to pay attention, and if there would be any withdrawal, even if just psychological, following treatment cessation.

## Reasoning

Reasoning refers to processes such as planning and decision making that simultaneously engage several cognitive domains, including memory, attention, cognitive control, and executive function. Complex reasoning is essential for carrying out complex tasks characteristic to humans, such as cooking a meal, writing an exam, or running a scientific experiment. Previous imaging studies have illustrated that psychedelics lead to the activation of regions involved in cognitive processes belonging to the higher band of complexity; for example in the case of playing the go/no-go task under the influence of LSD (Schmidt et al., 2018).

The literature on how reasoning is affected by psychedelics is scarce. In a study carried out by Quednow et al. (2012) psilocybin increased the number of errors on the Switching Stroop task in sixteen healthy volunteers, suggesting psilocybin impairs executive function. Another study using the Switching Stroop task, this time following ayahuasca administration in naturalistic settings, illustrated that accuracy is maintained under the influence even though the reaction time increases. This suggests cognitive control is a more demanding process under the influence of a psychedelic (Bouso et al., 2013), but also that the setting in which a study is carried out could account for a discrepancy in results. Concerning planning abilities, in the same naturalistic study carried out by Bouso et al. (2013) ayahuasca administration led to detrimental effects in the performance of the Tower of London planning task in less experienced subjects, but not in experienced ones; this begs a question about the extent to which prior psychedelic experience plays a role in the strength of acute drug effects on cognition. That is, can one learn to navigate the psychedelic state at optimal cognition? Another higher-order cognitive process studied, this time at average psychedelic doses, is inhibitory processing. LSD led to impaired performance on the go/no-go task in eighteen volunteers, which suggests that impulsivity is higher when under the influence (Schmidt et al., 2018).

Higher order cognitive processes have also been studied in the context of microdosing psilocybin and LSD. Granting the limited evidence available, microdosing appears to have unclear effects on reasoning. Prochazkova et al. (2018) illustrated that a microdose of psilocybin in naturalistic settings did not affect abstract reasoning as seen on performance on the Raven’s Matrix task– a commonly used method for assessing fluid intelligence in IQ tests—in 27 volunteers. In a different study, Bershad et al. (2019) illustrated that a microdose of LSD produced no effect on reasoning, as seen on the Digit Substitution task in twenty healthy volunteers. Contrastingly, Hutten et al. (2020) found that there was an impairment seen on the performance of this task under the effects of LSD at the low dose of 20 μg. Whilst Bershad et al. (2019) and Hutten et al. (2020) both conducted randomized double-blind placebo controlled within subjects studies on microdosing, the discrepancy in the results means that further research is needed to conclude whether microdosing impairs executive function, and if so what the threshold for this impairment is and what role individual differences play in driving these results.

It is very challenging to coherently interpret the current body of literature. The lack of replication with higher numbers of subjects, within similar experiential paradigms, under the influence of the same substance, and at similar dosages, advises future research to dedicate special attention to studying higher order cognition under the influence of psychedelics. From the limited studies available it is not unreasonable to conclude that a person under the influence of a psychedelic drug will have difficulties planning, take longer to exert cognitive control, and will be more impulsive. In order to establish validity, replicated studies with larger samples and extended experiments that include all psychedelics at different doses need to be carried out. These findings have strong implications considering that when unable to reason effectively, people cannot carry out complex tasks safely, efficiently, or rigorously.

## Emotional/Social Cognition

A human being is fundamentally a social being. By enabling effective communication, *social cognition* is critical to the functioning of an individual or group within a society (Young, 2008). The effects of classical hallucinogens on social cognition appear to be like those of traditional antidepressants and anxiolytics, but the studies pertaining to both classes of drugs still need to be replicated in larger trials (Rocha et al., 2019). The effects of psychedelics such as psilocybin and LSD on Emotional Faces Recognition have been reviewed by Preller and Vollenweider (2019) and Rocha et al. (2019). Previous studies illustrate that psilocybin led to connectivity changes during negative and positive facial emotion processing (Grimm et al., 2018), and that LSD and psilocybin reduce fear recognition on fMRI and EEG (Schmidt et al., 2013; Bernasconi et al., 2014; Dolder et al., 2016; Mueller et al., 2017) and enhance empathy and sociality (Dolder et al., 2016); this suggests psychedelics could influence social cognition.

The majority of studies looking at the effects of psychedelics on social cognition have investigated psilocybin. One study used seventeen subjects to shown that psilocybin impairs the recognition of negative facial emotions and enhances the recognition of positive facial expression, thus creating a bias for positive cues (Kometer et al., 2012). Pokorny et al. (2017) reported that psilocybin increased emotional empathy in 32 volunteers (as seen on the Multifaceted Empathy test) but not cognitive empathy. Interestingly, the 24 participants who were tested on the emotional dilemma task produced results unaffected by the influence of psilocybin; this signals that whilst empathy is enhanced, morality is not (Pokorny et al., 2017). More recently, increased emotional empathy to negative stimuli was observed after ayahuasca consumption compared to placebo post an ayahuasca ceremony (Uthaug et al., 2021b). Contrastingly, Kiraga et al. (2021) found an effect on cognitive empathy which was increased the day post an ayahuasca ceremony, but not on emotional empathy which was only increased a week post the ceremony. Other aspects of social cognition measured under psilocybin were social reward and social exclusion. Gabay et al. (2018) illustrated that psilocybin reduced the rejection of unfair options in nineteen participants playing the ultimatum game, and hypothesized that participants under the influence care more about social interaction itself rather than possible rewards. Preller et al. (2016) tested social exclusion under the influence of psilocybin in the context of the Cyberball task (which measures the amount of social neglect) and found that whilst participants felt less excluded from the social circle there was no difference in the amount of ball throws they received compared to placebo.

Studies looking at LSD illustrated that it increases emotional empathy (Dolder et al., 2016), specifically for positive facial cues (Pokorny et al., 2017), in a dose-dependent fashion. However, when testing LSD microdosing, no effects were present on affective rating using the Emotional Images Task (Bershad et al., 2019). When using the Cyberball task, LSD microdoses did not modulate the perceived number of received ball throws nor influence mood responses to rejection, which the authors attribute to the low dosage administered (Bershad et al., 2019). Only one study looking at the effect of ayahuasca on social cognition was carried out and illustrated that the drug had no effect on emotional face recognition in 22 volunteers (Rocha et al., 2021). However, researchers did present extensive hypotheses concerning why this might be the case—alkaloid degradation, learning effects, and education level, to name just a few.

Effects of psychedelic drugs on social cognition are of particular importance since they can influence how humans relate to one another, both in health and disease. A study carried out by Stroud et al. (2018) in seventeen depressed patients suggested that after an experience with psilocybin (combined with psychological support), patients had persistent improvements in facial recognition abilities; this also correlated with a decrease in anhedonia. Another study illustrated that psychedelic group experiences are beneficial to healthy subjects too, as per historical notes of group ceremonies (Kettner et al., 2021). Considering the available literature, we can infer that psychedelics minimize the feelings of social exclusion and make people more empathic; in other words, psychedelics appear to be beneficial to improving human relationships. However, they might also make people more susceptible to deceit and accepting unfair deals (Gabay et al., 2018).

## Creativity, Suggestibility and Language

Creativity and problem-solving have been regarded as critical abilities since the beginning of time. Creativity is a multilayered phenomenon, commonly defined as the ability to generate ideas, solutions, or products that are both novel and appropriate. The creative processes most extensively studied are convergent and divergent thinking. Convergent thinking requires identification of a single solution to a well-defined problem. Divergent thinking draws more on cognitive flexibility and the generation of multiple novel ideas (Mejia et al., 2021). The ability to think “outside of the box” has also been quoted to be affected in depression, anxiety, and other psychological disorders (Fresco et al., 2006; Forgeard and Elstein, 2014). It has been highlighted that the study of human creativity under the influence of psychedelics has been previously yielding inconclusive results, thus calling for contemporary methodologies to address this question (Sessa, 2008; Girn et al., 2020). Recent imaging studies have shown that networks associated with creative thinking are modulated during the psychedelic experience (Tagliazucchi et al., 2014; Mason et al., 2021). At the molecular level, 5-HT_2A_ agonism has been reported to be associated with enhanced cognitive flexibility (Clarke et al., 2004, 2007; Boulougouris et al., 2008; Kehagia et al., 2010) and improved associative learning (Harvey, 1996, 2003), but no study so far has linked psychedelic effects mediated by the 5-HT_2A_ receptor and enhanced creativity.

Creativity is often concomitantly examined with suggestibility. Only two studies looking at LSD’s effect on suggestibility have been conducted so far. The first study, which was limited to a small sample of ten healthy volunteers, showed that LSD enhances suggestibility on the Creative Imagination Scale, whilst cued imagery remained unaffected (Carhart-Harris et al., 2015). The second study showed that LSD increases adaptation to opinions expressed by a control group, but only if those opinions were not too different from participants’ own opinions (Duerler et al., 2020).

Microdosing psychedelics has been proposed as a means of enhancing creativity in healthy adults (Kuypers et al., 2019). A naturalistic study by Prochazkova et al. (2018) showed that a microdose of psilocybin can increase divergent thinking measured with the Alternative Uses Task, as well as convergent thinking measured with the Picture Concept Task. They also illustrated that an improvement in divergent thinking is marked by increased fluency, flexibility, and originality scores. Furthermore, they also note that reasoning illustrated by performance on the Raven’s Matrix task was not impacted at all, which suggests that creativity can be enhanced by psychedelics without affecting analytical thinking (Prochazkova et al., 2018). This is in line with early studies on the topic (Zegans et al., 1967). On the other hand, Mason et al. (2021) illustrated in a very elegant study that a normal dose of psilocybin impairs divergent thinking on the Alternative Uses task. Participants generated less ideas and associations, and had lower fluency and originality scores. They also had impaired performance on the Picture Concept task, suggesting poorer convergent thinking. Authors noted, however, that divergent thinking was increased once the dose wore off, but this was not the case for convergent thinking. This study suggests that the effects of psychedelics on creativity may persist past the acute phase of the experience. Interestingly (and of crucial importance), researchers highlight that psilocybin appeared to enhance the perceived quality of ideas generated despite impairing deliberate creative processes objectively (Mason et al., 2021). Conversely, microdosing LSD has been shown to have no effect on convergent thinking measured with the Remote Associations Task (Mednick, 1968) in 20 healthy volunteers (Bershad et al., 2019). Additionally, a study done by Kuypers et al. (2016) found that convergent thinking measured with the Picture Concept Task and divergent thinking measured with the Pattern/Line Meanings test decreased post ayahuasca administration in 26 healthy volunteers.

Language processing is an essential cognitive function concerning the detecting and comprehension of human speech. Language impairment is a hallmark of psychiatric disorders such as psychosis (Corcoran et al., 2020). Studies have shown there is a clear distinction between normal sober speech and speech under the influence of psychedelics, and that machine learning classifiers can distinguish between the two (Carrillo et al., 2018). The main question is what exactly makes speech under the influence of psychedelics different. Analysis of interviews from twenty volunteers undergoing an experience with LSD showed that the drug led to disorganized speech characterized by increased verbosity and reduced lexicon, similar to what is observed in schizophrenia (Sanz et al., 2021). Early studies showed that consistent use of LSD increased figurative speech in two out of three volunteers who used the drug over the course of a year and a half. This firstly indicates that the effects of LSD on language ability may persist even after the dose wears off, and secondly that there is a strong link between the effects of psychedelics on language processing and creativity (Natale et al., 1978). Previous research has also highlighted the link between the use of language and creative processes (Spitzer et al., 1996). Early on it was noted that psychedelics make speech less predictable and enhance free word associations (Amarel and Cheek, 1965). Furthermore, LSD in seven neurotic depressives caused individuals to make more personal statements and to use explanation and evaluations less often (Natale et al., 1978, 1979), which indicates that administration could be beneficial to the mental health of people undergoing talking therapies.

The problem of human creativity and psychedelics is multifactorial. Creativity, despite its common segregation into convergent and divergent thinking, remains a rather elusive process to study. Then, the effects of psychedelics are majorly dependent on context, since they may make people highly suggestible and heterogeneously impact creative processes. To date, there is no study assessing psychedelics and creativity in a natural environment. Whilst it is suggested that creativity might be enhanced under microdoses or post-experience with average doses, future studies would need to address the problem of environmental suggestibility when such tasks are carried out in laboratory settings. Another predominant problem is that of spontaneous insight and creative thought under the influence of psychedelics. Spontaneous insights are difficult to replicate under laboratory settings. That said, anecdotally, large doses of psychedelics have contributed to insights which have ultimately led to some of the biggest discoveries in modern science, such as PCR or the structure of DNA. It is understandable why in light of such stories, people working demanding jobs or in creative industries would be tempted to enhance their output with psychedelics.

## Limitations

Despite tremendous progress in reigniting the flame of psychedelics research and the positive results illustrated by early trials on mental health outcomes, our understanding of how psychedelics affect cognition in health or in disease is remarkably scarce. Studies are riddled with limitations that make it extremely difficult to integrate the findings in order to generate strong insights about the effects these drugs have on cognition. This problem is of one significant importance because these drugs have been proposed for a number of therapies (Siegel et al., 2021) and are increasingly used for both research (Oxford Analytica, 2021) and recreational purposes (Palamar and Le, 2018; Yockey et al., 2020). Policies are becoming progressively relaxed and more and more psychedelic clinics are opening up, selling the idea that psychedelic medicines are safe and reliable. It is correct to infer that 6–10 h long therapy sessions, already occurring in research, will become more ubiquitous—first in clinical trials and future data acquisition processes, then in psychedelic clinics, and eventually in everyday clinical practice. It has been noted that this rapid progress in already monetizing the therapies poses great risks, considering our extensive lack of knowledge. There is a need for additional research to continue challenging the outcomes observed to date (Rucker and Young, 2021).

One of the primary limitations in the study of psychedelic cognition is the difficulty researchers face in carrying out the research itself, namely problems with participants paying attention to or being engaged with the experiment—a fact noted extremely early on (Goldberger, 1966). Later it was illustrated that attention is indeed impaired at higher doses (Gouzoulis-Mayfrank et al., 2006; Daumann et al., 2008). The difficulty in setting up a study looking at cognition, as well as the length and demands that characterize classical cognitive assessment, could have deterred researchers from focusing on the acute effects psychedelics have on cognition. Efforts also need to be directed toward assessing all psychedelic drugs—including mescaline, which has been given very little attention—to facilitate comparison between different drug effects.

Where attempts have been made to carry out research on how psychedelics affect cognition acutely, the paradigms applied to test cognition differ widely across studies; this has unsurprisingly led to inconsistent results. For example, Carter et al. (2005) found no effects of psilocybin on working memory in eight human volunteers using the Spatial Span task from the CANTAB battery. But more recent research carried out by Barrett et al. (2018) found that in 20 healthy volunteers, psilocybin affected working memory when assessed with the Letter N-back task, as characterized by increased response time. Insufficient effort has been directed toward replicating previous findings with similar paradigms. Rather, new paradigms have been tested, which is why we are now at a stage where it is difficult to draw sweeping conclusions from existing studies. More recently citizen science online cognitive testing technologies (which employ yet to be validated computerized versions of classical neuropsychological assessments) such as Cognitron (for people taking psychedelics at any doses)^3^ and the Quantified Citizen app (for microdosing)^4^ have been used for testing how cognition is impacted by psychedelics in naturalistic settings.

Another problem observed in current studies is not assessing the effects of psychedelics on cognition at different timepoints in the trip with the same dosages, within the same sessions. The majority of the studies have only assessed effects at baseline and during the peak of the experience (roughly 2 h since administration). Research has shown how the intensity of effects vary during a trip, which suggests that the effects on cognition and perception would also vary. For example, Mason et al. (2021) illustrate that divergent thinking is impaired during a psilocybin experience, but once the dose wears off it increases compared to controls. This suggests that psychedelic effects are experience-timeline dependent, where effects are of impairment during the peak time and of enhancement in the afterglow state. There are no studies to date which consistently examine the same effects on cognition at baseline and then at different points throughout the psychedelic experience. A finer grain understanding of how psychedelics affect cognition is needed to establish the timepoints or doses where people are most vulnerable, and how exactly their vulnerabilities manifest.

Limitations in previous studies that make conclusions difficult to generalize have also to do with the different dosages of a psychedelic that the effects have been assessed at. It has been noted that for LSD there are different subjective effects at different dosages (Holze et al., 2020, 2021), and prior research illustrates that this is also the case with other drugs. For example, microdosing LSD has been shown to increase attention (Hutten et al., 2020), whilst higher doses of psychedelics such as DMT or psilocybin cause impairment (Carter et al., 2005; Gouzoulis-Mayfrank et al., 2006; Heekeren et al., 2007; Vollenweider et al., 2007; Daumann et al., 2008). Similarly, low doses of psilocybin have been shown to increase creative processes (Prochazkova et al., 2018), whereas higher doses cause acute impairment (Mason et al., 2021). Whilst it would be easy to conclude that high doses generally cause impairment and small microdoses generally cause enhancement, stricter experimentation and analysis is needed to establish the tipping point at which a psychedelic dose—potentially specific to each drug and/or entirely dependent on context—could cause impairment and which people are more likely to be affected.

Small sample sizes are the main culprits when considering limitations of the current studies on cognition. In the context of psychedelics this is particularly important since the experiences are subjective and by definition difficult to generalize (Preller and Vollenweider, 2018). Whilst in early studies it was acceptable to employ even just three volunteers per study (Natale et al., 1978, 1979), the twenty-first century sees an improvement, with the average number of subjects being 20–24 healthy volunteers. This increase in sample size, as well as the use of within subject study designs, means that studies benefit from sufficient statistical power to detect psychedelic-induced changes in cognition. Nevertheless, variations have been noted in how people perform on cognitive tasks under the influence of a psychedelic based on factors such as their drug use history (where more experienced users show better performances) (Bouso et al., 2013). Some studies have used naïve volunteers but others have limited participation to experienced and comfortable users (albeit in part due to safety guidelines). Future research needs to employ higher numbers of volunteers with different levels of experience and different baseline cognitive profiles to allow cross comparisons between sub-groups; this would provide us with a better understanding of the instances where cognition is positively or negatively affected by psychedelics. Moreover, most of the studies on cognition have employed healthy volunteers. Since psychedelics are proposed as treatments for neurological and psychiatric disorders, the lack of data in this field testing the cognition of volunteers suffering with various disorders *prior to the introduction of therapeutic licenses for these substances* is astonishing. Therefore, further efforts should be aimed at understanding the impact of moderating factors on the influence of psychedelics on cognition.

Although the existing research provides grounds for optimism there are still numerous gaps to be addressed in order to fully understand the impact and safety of these substances on individuals. Specifically, there is a question about the impact of these substances outside research laboratories, therapeutic settings, and other such controlled environments. It has been noted that set and setting does predict the responses to a psychedelic (Haijen et al., 2018). Controlled environments are lacking in the dynamism provided by the contexts wherein people have historically taken psychedelics, be it solitude within one’s own home, parties, religious ceremonies, or other social settings. There are numerous studies illustrating the impact of context on the psychedelic experience. Set and setting importance with regards with psychedelic (therapy) has been noted consistently (Carhart-Harris et al., 2016a; Hartogsohn, 2016, 2018; Carhart-Harris, 2018), and therapy rooms where psychedelic experiences are carried out in the presence of trained professionals are carefully designed to maximize comfort, which rarely matches the reality of casual psychedelic experiences. Moreover, in laboratory settings, throughout the experience, patients have their eyes closed and are offered support by highly trained (sober) individuals. Outside these contexts “sitters” might not always be available or even sought. Whilst the impact of set and setting is documented in the literature, very few studies report the effects of psychedelics in naturalistic settings. Given the significance of these very aspects to the outcomes of the experience (Haijen et al., 2018), extrapolating from findings within laboratory/therapeutic settings to predict outcomes of the psychedelic experience in naturalistic settings is not only scientifically incorrect but also dangerous. When it comes to cognition, effects that manifest as impairments in key cognitive domains could mean an inability to function that could be threatening to a person’s health. Therefore, it is necessary to understand how different substances impact different domains of cognition, at different doses, in different settings; to provide guidelines for designing harm reduction programs appropriately; and to educate the population prior to offering free access to psychedelic drugs.

## Future Directions

The field of psychedelic research offers a plethora of opportunities for scientific inquiry, and addressing the previously mentioned limitations is perhaps the most critical given that right now, safety guidelines are being developed, therapies are commencing, and recreational use is increasing. Rucker and Young (2021) also mention that it is unwise for so many clinics to be offering these therapies before psychedelics have been adequately tested and that this rush is undermining the credibility of the field. Humanity is racing toward psychedelics due to their obvious wow factor, diving headfirst into what seems to be the hope of psychiatry. But a repeat of the 60’s isn’t yet an impossibility. Extreme diligence is required in the way we carry out research and propose policies.

Addressing limitations posed by existing studies involves testing cognition with all classical psychedelic drugs suggested for therapeutic purposes. Further, the absence of data in relation to non-classical drugs often used recreationally, even though not yet suggested for therapy, should receive more attention—one such example being mescaline, whose impact on cognition has not been tested despite its recreational use being documented (Uthaug et al., 2021a). Paradigms need to be replicated with multiple substances, at multiple dosages, and with higher numbers of heterogeneous groups of participants. Importantly, these groups should include participants with diverse health conditions, and different levels of experience with psychedelics. This is particularly important since psychedelics are taunted as great and safe candidates for use in therapies. Furthermore, in order to facilitate our understanding of who is more likely to benefit from an enhancing effect from psychedelics—or alternatively, suffer from acute cognitive impairments—more research needs to focus on assessing how cognition is impacted at baseline levels in the same participants in a within-subject study design, rather than in comparison with completely different control samples. Even at microdoses, effects on attention suggest that there could be individual differences in all cognitive domains (Hutten et al., 2020). Importantly, we need also to investigate how cognition evolves at different times during the trip (as past studies have illustrated that effects can vary wildly from the acute stage to just after the trip) and follow up with cognitive tests to assess whether there are any sustained improvements or negative effects due to psychedelic use. Side-effects also need to be addressed. For instance, in some microdose studies there have been no effects on cognition (Family et al., 2020) but participants did report headaches, suggesting that psychedelics, even at lower doses, are not completely free of side effects. Headaches have been reported in other serotonergic hallucinogens, notably to occur in a dose-dependent manner with psilocybin exposure (Johnson et al., 2012). Conversely, there is evidence to suggest psychedelics can have beneficial effects on stopping cluster headaches (Sewell et al., 2006).

Going further, a unified approach to cognitive testing that is reproducible, scalable, validated against established cognitive measures, and addresses the identified limitations would be immensely beneficial in enhancing the body of knowledge about how psychedelics affect cognition in laboratory, clinical, and naturalistic settings.

## Author Contributions

The author confirms being the sole contributor of this work and has approved it for publication.

## Conflict of Interest

The author declares that the research was conducted in the absence of any commercial or financial relationships that could be construed as a potential conflict of interest.

## Publisher’s Note

All claims expressed in this article are solely those of the authors and do not necessarily represent those of their affiliated organizations, or those of the publisher, the editors and the reviewers. Any product that may be evaluated in this article, or claim that may be made by its manufacturer, is not guaranteed or endorsed by the publisher.

## Footnotes

1
https://pubmed.ncbi.nlm.nih.gov/

2
https://scholar.google.com/

3
https://psychedelics.cognitron.co.uk

4
www.microdose.me
