# Supplementary material for: Psychedelic Cognition—The Unreached Frontier of Psychedelic Science
Source: Front Neurosci. 2022 Mar 15;16:832375. doi: 10.3389/fnins.2022.832375 (PMC8989050; doi:10.3389/fnins.2022.832375)
Supplement: Supplementary file 1 [file Table_1.DOCX]

**Table 1.**

*Summary of literature addressing the acute effects of psychedelics on cognition*

| **Cognitive domain** | **Cognitive process** | **Type of study** | **Sample size** | **Cognitive assessment tool** | **Drug** | **Dose** | **Time of assessment** | **Effect observed** | **Reference** |
| --- | --- | --- | --- | --- | --- | --- | --- | --- | --- |
| Memory | Working memory | Counterbalanced double -blind placebo-controlled within subjects study | 8 | Spatial Span Task (CANTAB) | Psilocybin | 215 μg/kg | 120min after administration | No effect | *Carter et al., 2005* |
|  | Working memory | Counterbalanced double -blind Placebo-controlled within subjects study | 12 | Spatial Span Task (CANTAB) | psilocybin | 115 μg/kg, 250 μg/kg | Baseline, 100min since administration,  360min since administration | No effect at low dose, At high dose impairment 100min after administration compared to placebo | *Wittman et al., 2007* |
|  | Working memory | Observational within subjects study | 24 | Sternberg Task | ayahuasca | 100 ml tea // exact content of DMT not reported but confirmed via thin layer cromatography | Baseline, 120min after administration | impairment | *Bouso et al., 2013* |
|  | Working memory | Counterbalanced double -blind Placebo-controlled within subjects study | 19 | Letter N-back Task (CNB) | psilocybin | 10mg/70kg, 20mg/70kg,30 mg/70 kg | Baseline, 2 hours after administration | Dose dependent increased reaction time, but no effect on accuracy | *Barrett et al., 2018* |
|  | Working memory | Counterbalanced double -blind Placebo-controlled within subjects study | 20 | Free recall | psilocybin | 10mg/70kg, 20mg/70kg,30 mg/70 kg | Baseline, 2 hours after administration | Dose dependent impairment | *Barett et al., 2018* |
|  | Working memory | Counterbalanced double -blind  Placebo-controlled within subjects study | 20 | Dual N-back Task | LSD | 6.5 μg, 13 μg, 26 μg | 2 hours and 30 minutes after administration | No effect | *Bershad et al., 2019* |
|  | Working memory | Randomised double -blind Placebo-controlled within subjects study | 48 | Spatial Span Task (CANTAB) | LSD | 5 μg, 10 μg, 20 μg | Screening, baseline, 2-3h after administration, 4 weeks follow up | No effect | *Family et al., 2020* |
|  | Associative learning | Randomised double -blind Placebo-controlled within subjects study | 48 | Pair Associates Learning (CANTAB) | LSD | 5 μg, 10 μg, 20 μg | Screening, baseline, 2-3h after administration, 4 weeks follow up | No effect | *Family et al., 2020* |
| Attention | Attentional tracking | Counterbalanced double -blind placebo-controlled within subjects study | 8 | Multiple-Object Tracking | psilocybin | 215 μg/kg | 120min after administration | impairment | *Carter et al., 2005* |
|  | Inhibition of return | Randomised double -blind Placebo-controlled  crossover within subjects study | 15 | Covert Orienting of Attention Task | DMT | low DMT: a bolus injection of 0.15 or 0.2 mg/kg over 5 min followed by a break of 1 min, followed by continuous infusion with 0.01125 or 0.015 mg/kg min over 84 min, (2) high DMT: bolus injection of 0.2 or 0.3 mg/kg, break of 1 min and continuous infusion with 0.015 or 0.02 mg/kg min, | Baseline, 30 minutes into the continuous DMT infusion | Dose-dependent impairment | *Gouzoulis-Mayfrank et al., 2006* |
|  | Prepulse Inhibition | Randomised double -blind Placebo-controlled  crossover within subjects study | 15 | Acoustic startle | DMT | low DMT: a bolus injection of 0.15 or 0.2 mg/kg over 5 min followed by a break of 1 min, followed by continuous infusion with 0.01125 or 0.015 mg/kg min over 84 min, (2) high DMT: bolus injection of 0.2 or 0.3 mg/kg, break of 1 min and continuous infusion with 0.015 or 0.02 mg/kg min, | During the continuous DMT infusion | No effect | *Heekeren et al., 2007* |
|  | Prepulse Inhibition | Randomised double -blind Placebo-controlled within subjects study | 16 | Acoustic startle | psilocybin | 115μg/kg, 215 μg/kg, 315 μg/kg | 90 and 165 minutes after administration | Dose-dependent impairment of short (30 ms) PPI | *Vollenweider et al., 2007* |
|  | Sustained attention | Randomised double -blind Placebo-controlled within subjects study | 16 | Frankfurt Attention Inventory | psilocybin | 115μg/kg, 215 μg/kg, 315 μg/kg | 0, 105, 180 and 360 minutes after administration | impairment | *Vollenweider et al., 2007* |
|  | Inhibition of return | Randomised double -blind Placebo-controlled within subjects study | 14 | Covert Orienting of Attention Task | DMT | DMT: bolus injection of 0.15 mg/kg over 5 min followed by a break of 1 min, followed by continuous infusion with 0.01 up to 0.01875 mg/kg min over 20 min | During the continuous DMT infusion | impairment | *Daumann et al., 2008* |
|  | Prepulse Inhibition | Randomised double -blind Placebo-controlled within subjects study | 16 | Acoustic startle | psilocybin | 260 μg/kg | 60 minutes after administration | impairment | *Quednow et al., 2011* |
|  | Sustained attention | Randomised double -blind Placebo-controlled within subjects study | 24 | Psychomotor Vigilance Task | LSD | 0 μg, 5 μg, 10 μg, 20 μg | 1 day before administration, 180 min after administration | Enhancement at 5 and 20 μg, no effect at the other doses | *Hutten et al., 2020* |
|  | Visual attention | Randomised double -blind Placebo-controlled within subjects study | 48 | Rapid Visual Information Processing Task (CANTAB) | LSD | 5 μg, 10 μg, 20 μg | Screening, baseline, 120-180min after administration, 4 weeks follow up | No effect | *Family et al., 2020* |
| Reasoning | Executive function | Randomised double -blind Placebo-controlled within subjects study | 16 | Switching Stroop Task | psilocybin | 260 μg/kg | 85 minutes after administration | impairment | *Quednow et al., 2011* |
|  | Executive function | Observational within subjects study | 24 | Switching Stroop Task | ayahuasca | 100 ml tea // exact content of DMT not reported but confirmed via thin layer cromatography | Baseline, 120min after administration | Impaired reaction time but not accuracy | *Bouso et al., 2013* |
|  | Planning | Observational within subjects study | 24 | Tower of London | ayahuasca | 100 ml tea // exact content of DMT not reported but confirmed via thin layer cromatography | Baseline, 120min after administration | impairment | *Bouso et al., 2013* |
|  | Executive function | Counterbalanced double -blind Placebo-controlled within subjects study | 20 | Emotional Conflict Switching Stroop | psilocybin | 10mg/70kg, 20mg/70kg,30 mg/70 kg | Baseline, 120min after administration | No effect reported | *Barrett et al., 2018* |
|  | Inhibitory processing | Randomised double -blind Placebo-controlled within subjects study | 18 | Go/no-go Task | LSD | 100 μg | 200 min after administration | impairment | *Schmidt et al., 2018* |
|  | Abstract reasoning | Observational study | 38 | Raven’s Matrix Task | psilocybin | 0.22-0.44g of dried truffles depending on self-assessed body weight;  the 0.22g sample had on average 1557 μg/g psilocybin, the 0.33g sample had on average 1595 μg/g psilocybin and the 0.44g sample 1632 μg/g. | 90 minutes after self-administration | No effect | *Prochazkova et al., 2018* |
|  | Executive function | Counterbalanced double -blind Placebo-controlled within subjects study | 20 | Digit Substitution Task | psilocybin | 10mg/70kg, 20mg/70kg,30 mg/70 kg | Baseline, 120min after administration | Dose dependent impaired reaction time but not accuracy | *Barrett et al., 2018* |
|  | Executive function | Counterbalanced double -blind Placebo-controlled within subjects study | 20 | Digit Substitution Task | LSD | 6.5 μg, 13 μg, 26 μg | 160 minutes after administration | No effect | *Bershad et al., 2019* |
|  | Executive function | Randomised double -blind Placebo-controlled within subjects study | 24 | Digit Substitution Task | LSD | 0 μg, 5 μg, 10 μg, 20 μg | 1 day before administration, 160 minutes after administration | Impairment at 20 μg, no effect at the other doses | *Hutten et al., 2020* |
|  | Cognitive control | Randomised double -blind Placebo-controlled within subjects study | 24 | Cognitive Control Task | LSD | 0 μg, 5 μg, 10 μg, 20 μg | 1 day before administration, 160 minutes after administration | No effect | *Hutten et al., 2020* |
| Social cognition | Emotional discrimination | Randomised double -blind Placebo-controlled within subjects study | 17 | Reading the Mind in  the Eyes Test | psilocybin | 215 μg/kg | 130 min after administration | enhanced performance for positive  compared with negative items | *Kometer et al., 2012* |
|  | Emotional inhibition processing | Randomised double -blind Placebo-controlled within subjects study | 17 | Emotional go/no-go | psilocybin | 215 μg/kg | 130 min after administration | enhanced performance for positive  compared with negative items | *Kometer et al., 2012* |
|  | Social exclusion | Randomised double -blind Placebo-controlled  crossover within subjects study | 21 | Cyberball Task | psilocybin | 0.215 mg/kg | 75 minutes after administration | Feeling less excluded | *Preller et al., 2016* |
|  | Cognitive empathy | Randomised double -blind Placebo-controlled within subjects study | 32 | Multifaceted Empathy Test | psilocybin | 0.215 mg/kg | 160 minutes after administration | No effect | *Pokorny et al., 2017* |
|  | Emotional empathy | Randomised double -blind Placebo-controlled within subjects study | 32 | Multifaceted Empathy Test | psilocybin | 0.215 mg/kg | 160 minutes after administration | enhanced | *Pokorny et al., 2017* |
|  | Moral decision making | Randomised double -blind Placebo-controlled within subjects study | 24 | Emotional dilemma Task | psilocybin | 0.215 mg/kg | 160 minutes after administration | No effect | *Pokorny et al., 2017* |
|  | Social reward | Open label within subjects study | 19 | The Ultimatum Game | psilocybin | 2mg | Screening, 60 minutes after administration | Reduced rejection of unfair options | *Gabay et al., 2018* |
|  | Emotional discrimination | Pilot observational study in depressed patients | 17 | Dynamic Emotional Expression Recognition Task | psilocybin | 10mg (safety session), 25mg | Baseline, exact time after administration not reported, 1 week after administration | Enhancement (whereas depressed individuals in the study previously had a global deflicit in processing emotional faces) | *Stroud et al., 2018* |
|  | Social exclusion | Counterbalanced double -blind Placebo-controlled within subjects study | 20 | Cyberball Task | LSD | 6.5 μg, 13 μg, 26 μg | 160 minutes after administration | No effect | *Bershad et al., 2019* |
|  | Emotional discrimination | Counterbalanced double -blind Placebo-controlled within subjects study | 20 | Emotional Images Task | LSD | 6.5 μg, 13 μg, 26 μg | 160 minutes after administration | No effect | *Bershad et al., 2019* |
|  | Emotional discrimination | Pilot, proof-of-concept, randomised trial | 22 | Pictures of Facial Affect | ayahuasca | 1 mL/kg (1 µg DMT/mL) | 4 hours after administration, 1, 7, 14 and 21 days after administration and 3 months after administration | No effect | *Rocha et al., 2021* |
|  | Cognitive empathy | Observational study | 20 | Multifaceted Empathy Test | ayahuasca | 4 different average quantities depending on location (average 65.88mg, 69.7 mg, 19.20 mg, 75mg DMT per dose) | 1 day after ayahuasca ceremony | enhancement | *Kiraga et al., 2021* |
|  | Cognitive empathy | Observational study | 19 | Multifaceted Empathy Test | ayahuasca | 4 different average quantities depending on location (average 65.88mg, 69.7 mg, 19.20 mg, 75mg DMT per dose) | 7 days after ayahuasca ceremony | enhancement | *Kiraga et al., 2021* |
|  | Emotional empathy (implicit) | Observational study | 19 | Multifaceted Empathy Test | ayahuasca | 4 different average quantities depending on location (average 65.88mg, 69.7 mg, 19.20 mg, 75mg DMT per dose) | 1 day after ayahuasca ceremony, 7 days after ayahuasca ceremony | Enhancement 7 days after the ceremony | *Kiraga et al., 2021* |
|  | Cognitive empathy | Placebo-controlled study  within subjects | 14 | Multifaceted Empathy Test | ayahuasca | 7 capsules (mean DMT concentration 3.6±0.2 mg/g) or 1 cup brew unknown dosage | Post-ayahuasca ceremony | No effect | *Uthaug et al., 2021b* |
|  | Emotional empathy | Placebo-controlled study  within subjects | 14 | Multifaceted Empathy Test | ayahuasca | 7 capsules (mean DMT concentration 3.6±0.2 mg/g) or 1 cup brew unknown dosage | Post-ayahuasca ceremony | enhancement | *Uthaug et al., 2021b* |
| Suggestibility | Suggestibility | Placebo controlled within subjects | 10 | Creative Imagination Scale | LSD | 40–80 μg (one received 40 μg, two 50 μg, six 70 μg and one 80 μg) | exact time after administration not reported | enhancement | *Carhart-Harris et al., 2015* |
|  | Cued imagery | Placebo controlled within subjects | 10 | Mental Imagery Test | LSD | 40–80 μg (one received 40 μg, two 50 μg, six 70 μg and one 80 μg) | exact time after administration not reported | No effect | *Carhart-Harris et al., 2015* |
|  | Social adaptation to other’s opinions | Randomised double-blind, placebo-controlled, parallel-group study | 24 | Social influence paradigm | LSD | 100 µg | 330 min after administration | Enhancement in specific cases | *Duerler et al., 2020* |
| Creativity | Convergent and Divergent thinking | Observational within subjects study | 26 | Picture Concept Task | ayahuasca | Group 1: average ± SD total volume of ayahuasca of 116.7 ± 17.1 ml with 0.65 mg/ml DMT, Group 2: 44.5 ± 15.6 ml with 0.96 mg/ml DMT. Participants received a top-up dose if desired (by the participant) | Baseline (3h before the ayahuasca session), 90-120 minutes after the first dose, after the second dose | Impairment of convergent thinking, enhancement of divergent thinking | *Kuypers et al., 2016* |
|  | Divergent thinking | Observational within subjects study | 26 | Pattern/Line Meanings test | ayahuasca | Group 1: average ± SD total volume of ayahuasca of 116.7 ± 17.1 ml with 0.65 mg/ml DMT, Group 2: 44.5 ± 15.6 ml with 0.96 mg/ml DMT. Participants received a top-up dose if desired (by the participant) | Baseline (3h before the ayahuasca session), 90-120 minutes after the first dose, after the second dose | No effect | *Kuypers et al., 2016* |
|  | Divergent thinking | Observational Study | 33 | Alternative Uses Task | psilocybin | 0.22-0.44g of dried truffles depending on self-assessed body weight;  the 0.22g sample had on average 1557 μg/g psilocybin, the 0.33g sample had on average 1595 μg/g psilocybin and the 0.44g sample 1632 μg/g. | 90 minutes after self-administration | enhancement | *Prochazkova et al., 2018* |
|  | Convergent thinking | Observational Study | 27 | Picture Concept Task | psilocybin | 0.22-0.44g of dried truffles depending on self-assessed body weight;  the 0.22g sample had on average 1557 μg/g psilocybin, the 0.33g sample had on average 1595 μg/g psilocybin and the 0.44g sample 1632 μg/g. | 90 minutes after self-administration | enhancement | *Prochazkova et al., 2018* |
|  | Convergent thinking | Counterbalanced Double-blind Placebo-controlled within subjects study | 20 | Remote Associations Task | LSD | 6.5 μg, 13 μg, 26 μg | 160 minutes after administration | No effect | *Bershad et al., 2019* |
|  | Divergent thinking | Randomised double-blind, placebo-controlled, parallel-group study | 60 | Alternative Uses task | psilocybin | 0.17 mg/kg | Baseline, 130 minutes after administration, 7 days after administration | impairment | *Mason et al., 2021* |
|  | Convergent thinking | Randomised double-blind, placebo-controlled, parallel-group study | 60 | Picture Concept task | psilocybin | 0.17 mg/kg | Baseline, 120 minutes after administration, 7 days after administration | impairment | *Mason et al., 2021* |
| Language processing | Language ability | Observational study in 10 alcohol use disorder patients | 10 | 100-word samples of spontaneous speech | LSD | 100-200 μg | Randomly sampled speech post administration | Decreased number of words spoken, Enhanced free word association | *Amarel and Cheek 1965* |
|  | Language ability | Randomised Double-blind Placebo-controlled within subjects study in depressed patients | 3 | 5 min monologues | LSD | 50–200 μg | Baseline, after the drug effects passed | Increased use of personal statements, less use of evaluation | *Natale et al., 1978* |
|  | Speech organisation | Randomised Double-blind Placebo-controlled within subjects study | 20 | Computational analysis of interviews | LSD | 75 μg | Approximately 285 min after administration | Disorganised speech (higher verbosity but reduced lexicon) | *Sanz et al., 2021* |
